# Quantitative Evaluation of Transtibial Aiming Accuracy in Anterior Cruciate Ligament Reconstruction

**DOI:** 10.7759/cureus.95763

**Published:** 2025-10-30

**Authors:** Anastasios G Roustemis, Dimitrios Koulalis, Dimitrios S Mastrokalos

**Affiliations:** 1 First Orthopaedic Department, Attikon Hospital, National and Kapodistrian University of Athens School of Medicine, Athens, GRC

**Keywords:** acl reconstruction, anteromedial portal, femoral tunnel placement, quantitative accuracy, transtibial technique

## Abstract

Background

Precise femoral tunnel placement is a critical determinant of anterior cruciate ligament (ACL) reconstruction success. This study aimed to evaluate the accuracy of transtibial aiming in relation to a predrilled anatomic femoral tunnel using radiographic and intraoperative photographic measurements.

Methodology

In total, 43 single-bundle ACL reconstructions with hamstring autografts were analyzed. After drilling the femoral tunnel through the anteromedial portal, a 4-mm offset guide was inserted transtibially toward its center. Tibial tunnel angles were measured in coronal and sagittal planes, and the absolute distance (D) between the centers of the femoral tunnel and the transtibial K-wire was recorded. A normalized index (I = D/radius, R) was calculated, with I <1 indicating that the transtibial wire remained within the tunnel perimeter.

Results

The mean femoral tunnel diameter was 7.44 mm (R = 3.72 mm), mean D was 4.50 mm (1.75-6.85 mm), and the overall mean index was 1.2. For tunnels of 7.0 and 7.5 mm, the mean index exceeded 1, while for tunnels of 8.0-9.0 mm, it was less than 1. Deviation showed no correlation with tibial tunnel angulation. In no case did the transtibial K-wire reach the exact femoral center.

Conclusions

The findings of this study demonstrated that transtibial aiming lacks sufficient precision for anatomic femoral tunnel placement, supporting the anteromedial portal approach as the more accurate technique.

## Introduction

The anterior cruciate ligament (ACL) is the primary stabilizer against anterior tibial translation and rotational displacement of the lateral tibial condyle [1,2]. Biomechanical studies have demonstrated that the anteromedial bundle primarily limits anterior tibial translation in flexion, whereas the posterolateral bundle resists anterior translation near extension and contributes significantly to rotational stability [1,2]. In recent years, there has been growing interest in double-bundle reconstruction to restore both translational and rotational stability [3,4]. However, creating two femoral tunnels is technically demanding and associated with greater bone trauma. Consequently, single-bundle reconstructions with posteriorly or horizontally oriented femoral tunnels have been proposed as a compromise, aiming to mimic the function of the posterolateral bundle [5].

Among the available techniques, femoral tunnel preparation may be performed transtibially or via the anteromedial portal. The latter allows unconstrained and more anatomic placement, and is widely regarded as the preferred technique for double-bundle and all-inside reconstructions [6]. In contrast, the transtibial approach is limited by the orientation of the tibial tunnel, which constrains the femoral entry point and may compromise accuracy [7].

We have previously reported on this patient cohort, demonstrating that transtibial aiming often fails to achieve anatomic femoral placement when assessed categorically as being within, at the perimeter, or outside the tunnel [8]. While that study highlighted the limited accuracy of the transtibial approach, it did not quantify the magnitude of deviation. The present work builds on those findings by applying a quantitative methodology using radiographic and intraoperative photographic documentation. Specifically, we measured the absolute distance (D) between the transtibial K-wire and the femoral tunnel center, and introduced a normalized index (I = D/radius of the femoral tunnel) to account for tunnel size. This metric-based approach offers a more precise understanding of the geometric limitations of transtibial aiming in single-bundle ACL reconstruction.

## Materials and methods

A total of 43 patients (43 knees) underwent ACL reconstruction using hamstring autografts with an implant-free press-fit technique, as previously described [9,10]. All procedures were performed by the same surgeon (H.H.P.) between 2004 and 2005. After diagnostic arthroscopy and treatment of concomitant lesions, the semitendinosus and gracilis tendons were harvested and prepared as a quadrupled graft for single-bundle reconstruction.

**Figure 1:**
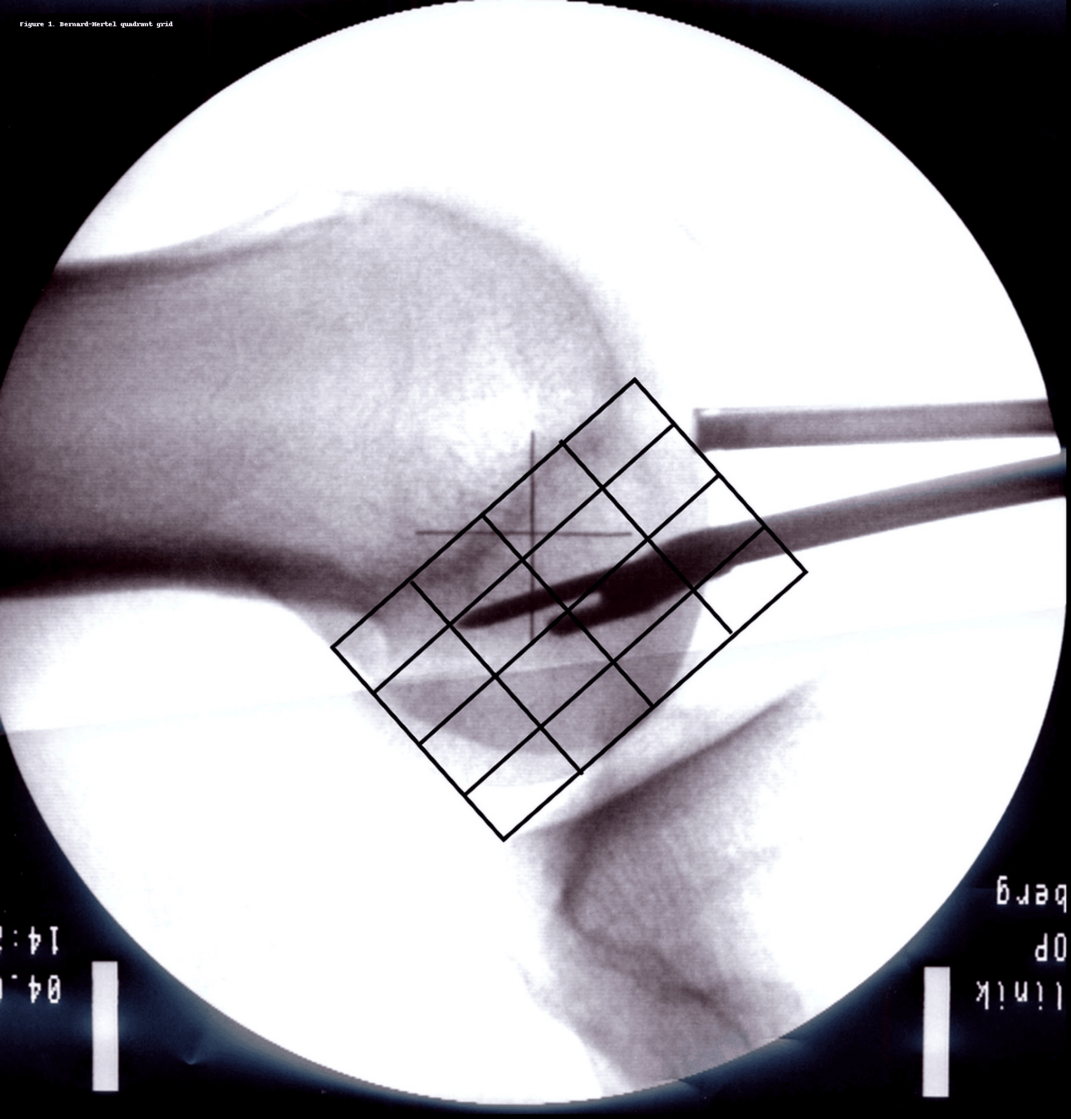
Fluoroscopic image of the lateral femoral condyle with the Bernard-Hertel quadrant grid used for femoral tunnel localization.

Femoral tunnel preparation was performed under anterolateral portal visualization. After resection of ACL remnants, a 4-mm offset guide was introduced through the anteromedial portal at 90° of flexion, aiming at the 09:30 (right knee) or 14:30 (left knee) clock-face position. A K-wire was placed, confirmed fluoroscopically [9], and referenced to the Bernard-Hertel quadrant method [11] (Figure 1). The knee was flexed to 120°, and the tunnel was completed with a drill bit matching the graft diameter.

**Figure 2:**
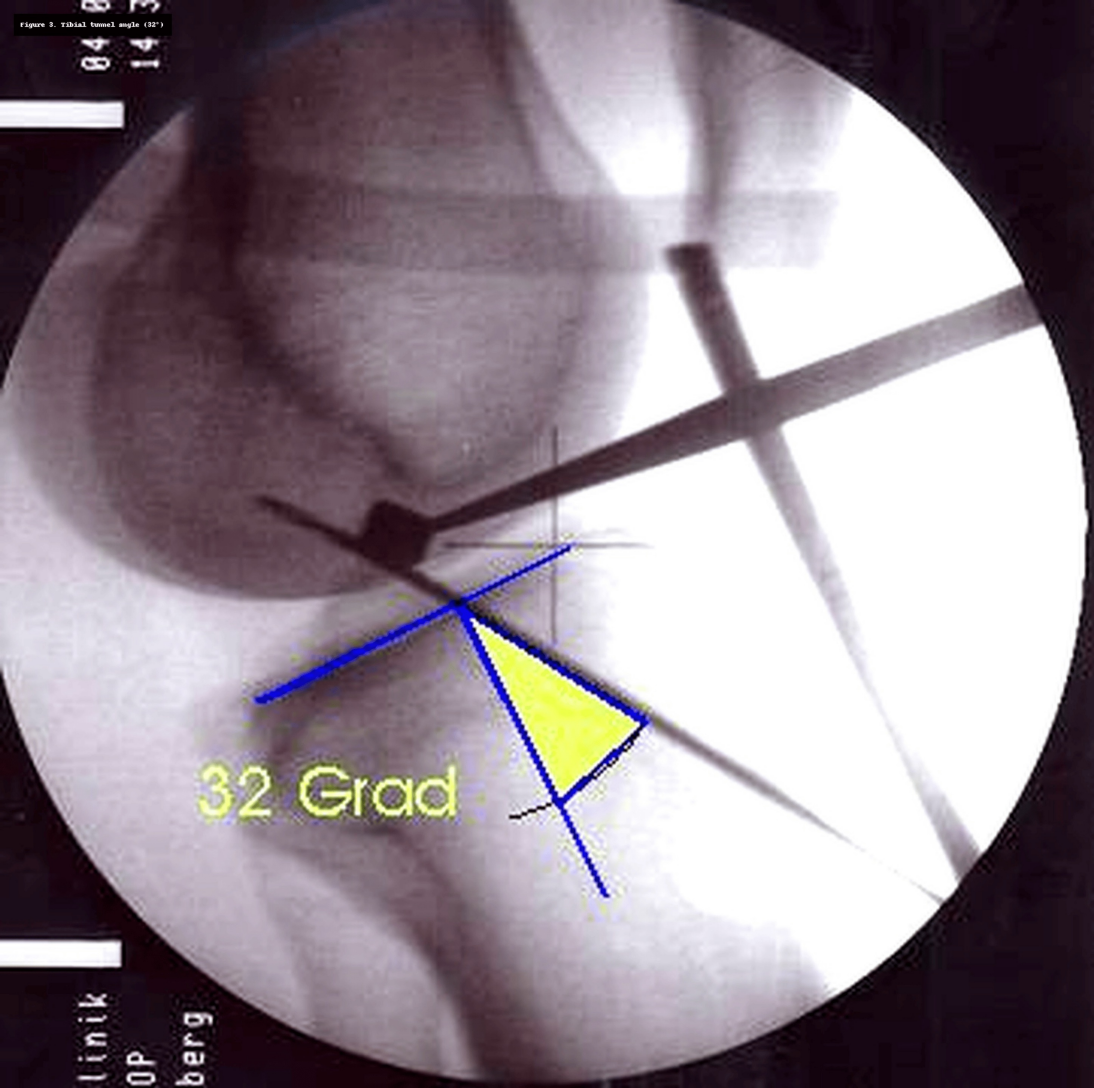
Radiographic measurement of the tibial tunnel angle in the sagittal plane (32°).

The tibial tunnel was drilled with the guide angled at 50°, positioned 1 cm above the pes anserinus and 1.5-2 cm medial to the tibial tubercle [12,13]. Aiming for approximately 65° in both coronal and sagittal planes [14,15], the K-wire was directed along the posterior rim of the anterior horn of the lateral meniscus toward the medial tibial spine [9,16]. Placement was documented radiographically (Figure 2), and impingement was excluded intraoperatively.

**Figure 3:**
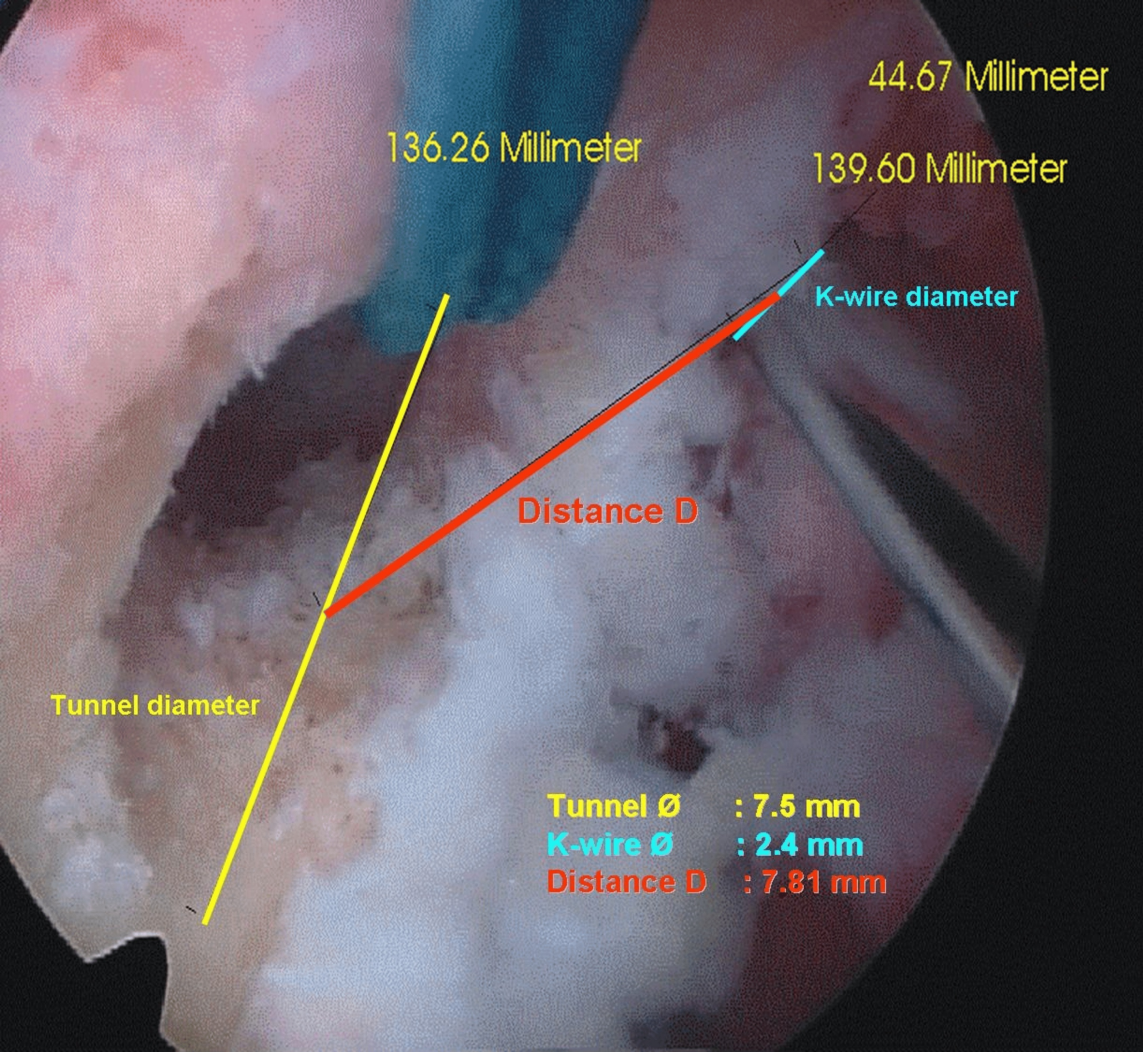
Arthroscopic photograph demonstrating measurement of the distance (D) between the center of the femoral tunnel and the transtibial K-wire. Calibration was performed using tunnel and K-wire diameters.

Transtibial aiming was then attempted with the 4-mm guide to reach the center of the predrilled femoral tunnel, and the position of the K-wire was documented with arthroscopic images (Figure 3). Reconstruction was completed by press-fit graft fixation.

Two independent observers, blinded to patient identity, evaluated anonymized image sets twice, including tibial tunnel radiographs and arthroscopic views. Measurements included tibial tunnel angles in coronal and sagittal planes (Figure 2), as well as the absolute distance (D) between the centers of the femoral tunnel and the transtibial K-wire (Figure 3). Calibration was performed using the known K-wire diameter (2.4 mm). A normalized index was then calculated (I = D/tunnel radius), where I = 0 indicated exact centering, I <1 placement within the tunnel, and I >1 placement outside.

Continuous variables were reported as mean, standard deviation, standard error of the mean, and sample size. Categorical variables were summarized as frequencies and percentages. Intraobserver and interobserver reliability for continuous variables was assessed with paired t-tests, while categorical agreement was evaluated using the Kappa statistic and McNemar’s test. Comparisons between groups were performed using Fisher’s exact test, with p-values <0.05 considered significant. Statistical analyses were conducted using SPSS version 10.0 (SPSS Inc., Chicago, IL, USA).

## Results

Intraobserver reliability was high for the first observer, with minimal deviation between repeated measurements (p = 0.694). The second observer demonstrated greater variability between first and second measurements (p = 0.05), and agreement was significantly lower compared to the first observer on the second measurement (p = 0.007).

The tibial tunnel center was located at a mean of 41.2% ± 3.4% (range = 34-46.6%) of the anteroposterior tibial plateau depth. The mean femoral tunnel diameter was 7.44 ± 0.6 mm (range = 7-9 mm), corresponding to a mean radius of 3.72 mm. Distribution by size included 21 tunnels of 7.0 mm, 11 tunnels of 7.5 mm, seven tunnels of 8.0 mm, three tunnels of 8.5 mm, and one tunnel of 9.0 mm.

The mean distance (D) between the transtibial K-wire and the femoral tunnel center was 4.50 ± 1.5 mm (range = 1.75-6.85 mm). The mean tibial tunnel angle was 26.1° ± 2.8° (range = 22-34°) in the anteroposterior plane and 25.7° ± 2.6° (range = 22-34°) in the sagittal plane.

In the anteroposterior view, the mean D was 4.2 ± 1.7 mm for tibial tunnel angles <26.5° and 4.8 ± 1.5 mm for angles ≥26.5° (p = 0.325). In the sagittal view, the mean D was 4.55 ± 1.6 mm for tibial tunnel angles <25.8° and 4.52 ± 1.3 mm for angles ≥25.8° (p = 0.716).

When expressed as the normalized index (I = D/R), tunnels of 8.0 mm diameter showed significantly different mean D compared with tunnels of 7.0 mm (p = 0.025) and 7.5 mm (p = 0.018). For tunnels of 7.0 mm (n = 21) and 7.5 mm (n = 11), the mean index values were 1.19 and 1.18, respectively, corresponding to placement outside the tunnel perimeter. For the remaining 11 (8.0-9.0 mm) cases, the mean index was <1, indicating placement within the tunnel perimeter. A representative case of a 7.5-mm tunnel with I >1 is shown in Figure 3. The overall mean index across all patients was 1.20 (Table 1).

**Table 1:** Femoral tunnel radius, mean distance (D), and index (I).

Number of cases	Tunnel radius (mm)	Distance D (mean ± SD, mm)	Minimum–Maximum (mm)	Index (I)
21	3.5 (Ø 7.0 mm)	4.16 ± 1.6	1.75–7.42	1.19
11	3.75 (Ø 7.5 mm)	4.43 ± 1.3	2.86–6.36	1.18
7	4.0 (Ø 8.0 mm)	3.92 ± 1.5	2.50–6.66	0.98
3	4.25 (Ø 8.5 mm)	4.53 ± 1.4	2.79–6.71	0.95
1	4.5 (Ø 9.0 mm)	2.20 ± 0.0	—	0.48
Total 43	3.72 ± 0.3	4.50 ± 1.5	1.75–6.85	1.20

## Discussion

In this secondary analysis of the same cohort previously reported in SICOT-J, we focused specifically on the quantitative accuracy of transtibial aiming using the parameters of distance (D) and index (I) [8]. While our earlier publication emphasized the overall inaccuracy of transtibial aiming, the present study provides a more detailed geometric evaluation that allows a better understanding of the magnitude of deviation [8]. We found that in no case did the transtibial K-wire reach the exact center of the femoral tunnel, with a mean deviation of 4.5 ± 1.5 mm (range = 1.75-6.85 mm). The mean index (I) was 1.2, indicating that in most cases the transtibial trajectory resulted in a position outside the femoral tunnel perimeter. Larger femoral tunnel diameters were associated with relatively lower indices, suggesting that increased tunnel size provided greater tolerance for guide manipulation and a slightly higher probability of remaining within the tunnel footprint. However, no correlation was observed between D and tibial tunnel angles in either the coronal or sagittal plane, and patient-related variables such as age and sex were not associated with deviation. These findings underline that the technical limitations of the transtibial approach are primarily geometric, rather than patient-dependent.

Our results are consistent with previous biomechanical and clinical observations describing the limitations of the transtibial technique. Ristanis et al. (2005) [3] demonstrated that the constrained trajectory of the tibial tunnel prevents reliable access to the native femoral footprint, while Kaseta et al. (2008) [17] emphasized that the offset between the femoral guide and the tibial tunnel radius further compromises accuracy. In our series, the majority of deviations exceeded the femoral tunnel radius, meaning that the K-wire was typically positioned outside the anatomical insertion site, often in a deeper and more vertical orientation. This is clinically relevant, as non-anatomic positioning may lead to altered graft isometry, higher graft tension in flexion, and potentially increased risk of failure. Recent comparative imaging studies confirm that conventional transtibial drilling tends to create more vertical and anterior tunnels compared to anteromedial portal or outside-in approaches, and rarely achieves the center of the native ACL footprint [18].

The pursuit of anatomic ACL reconstruction has driven the development of double-bundle techniques, which attempt to restore the functions of both the anteromedial and posterolateral bundles [5,19]. While conceptually attractive, double-bundle reconstructions are technically demanding, require two femoral tunnels, and are associated with greater bone loss and prolonged operative times. Comparative clinical studies have shown minimal differences between single- and double-bundle reconstructions when the femoral tunnel is placed more posteriorly and horizontally to mimic the posterolateral bundle [20,21]. Biomechanical studies support that femoral tunnels positioned at or beyond the 10 o’clock position improve resistance to rotational loads, though this may come at the expense of anterior stability [22,23]. Clinical outcomes, including International Knee Documentation Committee and pivot shift scores, remain inconsistent across techniques, with some authors reporting equivalence when single-bundle grafts are oriented more laterally [2,24,25]. More recently, modified transtibial double-bundle techniques have been proposed to overcome the geometric restrictions of the classic transtibial approach. Using careful guide manipulation and partially overlapping tibial tunnels, these modifications have achieved anatomic tunnel placement in over 90% of cases on postoperative 3D-CT [26]. However, nonanatomic outliers and posterior wall blowouts still occur, particularly in heavier patients or those with low tibial posterior slope, underscoring the technical challenges of consistently reproducing anatomic positions through the transtibial route [26].

The anteromedial portal approach has gained widespread acceptance because it allows unconstrained access to the femoral footprint and avoids many of the mechanical limitations inherent to the transtibial technique [6,27]. Our findings support this notion: despite variation in tibial tunnel orientation, transtibial aiming did not reliably achieve the femoral center. In addition, recent imaging-based modeling has shown that femoral tunnel length is highly dependent on coronal and sagittal drilling angles. The AM portal technique, when performed at lower obliquity angles, may create shorter tunnels (<35 mm), which could compromise graft-to-bone healing, especially in female patients with smaller bone dimensions [28]. Conversely, obliquity angles ≥45° generate femoral tunnels of adequate length, comparable to those obtained with the transtibial technique, thereby combining anatomic placement with sufficient tunnel length for secure graft fixation [28]. This highlights that both the accuracy of footprint targeting and tunnel length must be considered when choosing between techniques.

This study has several limitations. Measurements of distances and angles from radiographs and arthroscopic images are inherently subject to variability, as they are based on 2D representations of 3D structures, although intra- and interobserver reliability were acceptable. The sample size was moderate, and all reconstructions were performed by a single surgeon, which may limit external generalizability. Furthermore, clinical outcomes such as knee stability, patient-reported scores, or graft survival were not assessed in this analysis, as the primary focus was to evaluate geometric accuracy. Despite these limitations, the application of quantitative metrics such as D and I provides additional insights that complement our previous categorical analysis and offer a more precise demonstration of the geometric constraints of transtibial aiming.

By showing that transtibial aiming consistently deviates from the anatomic femoral center, this study adds to the growing body of evidence that supports the use of the anteromedial portal as the preferred method for femoral tunnel creation in single-bundle ACL reconstruction. Nevertheless, modified transtibial techniques may offer a partial solution by improving the reproducibility of anatomic placement, while careful attention to tunnel orientation is necessary to preserve adequate tunnel length and optimize graft healing [26,28].

## Conclusions

Transtibial aiming did not achieve central femoral tunnel placement in any case of this cohort. Quantitative analysis demonstrated a mean deviation of 4.5 mm and a mean index of 1.2, indicating that most transtibial trajectories fell outside the tunnel perimeter. Although larger femoral tunnels modestly improved accuracy, tibial tunnel angles and patient-related factors did not influence deviation. These findings highlight the geometric limitations of the transtibial technique and reinforce the anteromedial portal approach as the more reliable method for achieving anatomic femoral tunnel positioning in single-bundle ACL reconstruction.
